# Establishment and validation of the survival prediction risk model for appendiceal cancer

**DOI:** 10.3389/fmed.2022.1022595

**Published:** 2022-10-28

**Authors:** Tao Liu, Junli Mi, Yafeng Wang, Wenjie Qiao, Chenxiang Wang, Zhijun Ma, Cheng Wang

**Affiliations:** ^1^Department of Gastrointestinal Oncology, Qinghai University Affiliated Hospital, Xining, China; ^2^The Graduate School of Qinghai University, Xining, China

**Keywords:** gastrointestinal surgery direction, appendicular cancer, prognosis, Lasso regression, tumor, treatment

## Abstract

**Objective:**

Establishing a risk model of the survival situation of appendix cancer for accurately identifying high-risk patients and developing individualized treatment plans.

**Methods:**

A total of 4,691 patients who were diagnosed with primary appendix cancer from 2010 to 2016 were extracted using Surveillance, Epidemiology, and End Results (SEER) ^*^ Stat software. The total sample size was divided into 3,283 cases in the modeling set and 1,408 cases in the validation set at a ratio of 7:3. A nomogram model based on independent risk factors that affect the prognosis of appendix cancer was established. Single-factor Cox risk regression, Lasso regression, and multifactor Cox risk regression were used for analyzing the risk factors that affect overall survival (OS) in appendectomy patients. A nomogram model was established based on the independent risk factors that affect appendix cancer prognosis, and the receiver operating characteristic curve (ROC) curve and calibration curve were used for evaluating the model. Survival differences between the high- and low-risk groups were analyzed through Kaplan–Meier survival analysis and the log-rank test. Single-factor Cox risk regression analysis found age, ethnicity, pathological type, pathological stage, surgery, radiotherapy, chemotherapy, number of lymph nodes removed, T stage, N stage, M stage, tumor size, and CEA all to be risk factors for appendiceal OS. At the same time, multifactor Cox risk regression analysis found age, tumor stage, surgery, lymph node removal, T stage, N stage, M stage, and CEA to be independent risk factors for appendiceal OS. A nomogram model was established for the multifactor statistically significant indicators. Further stratified with corresponding probability values based on multifactorial Cox risk regression, Kaplan–Meier survival analysis found the low-risk group of the modeling and validation sets to have a significantly better prognosis than the high-risk group (*p* < 0.001).

**Conclusion:**

The established appendix cancer survival model can be used for the prediction of 1-, 3-, and 5-year OS and for the development of personalized treatment options through the identification of high-risk patients.

## Introduction

Appendiceal cancer is a rare malignancy of the digestive tract with an approximate incidence of 0.2–0.5% (1, 2). It has an occult onset, a lack of specificity of early symptoms, and there is difficulty in anatomically locating the right adnexal mass in women (3). At the same time, a lack of understanding of appendix cancer among clinicians often results in misdiagnosis and missed diagnosis, causing great difficulties in terms of clinical treatment and seriously affecting the prognosis of patients. In addition, as a result of the low incidence of appendiceal cancer, there is insufficient clinical attention, and appendix cancer studies relating to prognosis have mainly been based on small sample size, single-center analysis, and a lack of strong evidence, resulting in a certain bias. Therefore, based on the large sample size of the SEER database, this study screens the best variables using Lasso regression, excludes some repeated and unnecessary parameters, and solves the overfitting problem. Finally, the nomogram model is established, and high-risk groups are further stratified by risk factors as a basis for prognosis improvement.

## Materials and methods

### Data collection

The Surveillance, Epidemiology, and End Results (SEER) database is publicly available and covers ~35% of the US population. Clinical data from SEER ^*^, including patient age, race, sex, pathological type, pathological stage, surgery, radiotherapy, chemotherapy, lymphadenectomy number, insurance status, marital status, T stage, N stage, M stage, tumor size, and CEA from 2010 to 2016 was downloaded using Stat software. A total of 4,691 patients were ultimately included.

### Inclusion criteria

Inclusion criteria were as follows: (1) Pathological diagnosis of primary appendiceal cancer; (2) complete clinicopathological data.

### Exclusion criteria

Exclusion criteria were as follows: (1) Lack of patient follow-up information; (2) unknown or missing general information and data; (3) combined with other malignant or non-primary tumors.

### Statistical treatment

SPSS 25.0 software was used for performing statistical analysis of the data. The count data were expressed as n (%), and a comparison between groups was performed using the χ^2^-test. Univariate cox risk regression analysis of risk factors influencing appendiceal cancer overall survival (OS). Based on R3.6.3 the software further screened the best variables through the incorporation of statistically significant single-factor Cox risk regression into Lasso regression and cross-validation and the final selected variables into multifactor Cox risk regression as a means of determining age, pathological stage, surgery, lymph node removal number, T stage, N stage, M stage, and CEA independent risk factors that affect the prognosis of appendix cancer. The multivariate Cox regression index with statistical significance was used for establishing a nomogram model with R software, and the ROC curve and calibration curve were further drawn in order to evaluate model reliability. Finally, probabilistic values were calculated based on multifactorial Cox risk regression, the optimal cut-off that corresponds to the maximum Jordan index of the ROC curve was divided into high-risk and low-risk groups, and the survival differences between the appendix cancer modeling and validation sets were calculated by Kaplan–Meier survival analysis and log-rank assays. *P* < 0.05 was considered to be significant.

### Ethics and consent

The authors were authorized to extract data from the SEER study by the National Cancer Institute. Access to data *via* the SEER database requires no informed patient consent (SEER ID: 13846—Nov2020). This study is a retrospective analysis that is in strict compliance with the Helsinki Declaration of 1964 and subsequent amendments or similar ethical standards.

## Results

### Comparison of the pathological characteristics of patients in the appendiceal cancer modeling and validation sets

A total of 4,691 appendix cancer cases were included in this study, including 3,283 appendix cancer modeling sets and 1,408 appendix cancer validation sets, whereby 2,107 patients were male (44.9%) and 2,584 were female (55.1%). A comparison between the two groups identified significant differences in race, pathological type, and insurance (*p* < 0.05). Age, race, pathological type, pathological stage, surgery, radiotherapy, chemotherapy, number of lymphadenectomies, T stage, N stage, M stage, tumor size, and CEA were found to not be significantly different (*p* > 0.05), as shown in Table 1.

**Table 1:** Analysis of clinical case data for appendix cancer.

	**Training cohort (*n* = 3,283)**	**Validation cohort (*n* = 1,408)**	***X* ^2^**	***P*-value**
**Age**			2.812	0.094
<60	1,825 (55.6%)	820 (58.2%)		
≥60	1,458 (44.4%)	588 (41.8%)		
**Race**				
White people	2,724 (83.0%)	1,181 (83.9%)	95.82	<0.001
Black people	293 (8.9%)	204 (14.5%)		
Other	266 (8.10%)	23 (1.6%)		
**Sex**			0.445	0.505
Man	1,485 (45.2%)	622 (44.2%)		
Woman	1,798 (54.8%)	786 (55.8%)		
**Pathology type**			10.965	0.012
Carcinoid	720 (21.9%)	308 (21.9%)		
Cup-shaped cell Carcinoma	334 (10.2%)	182 (12.9%)		
Adenocarcinoma	456 (13.9%)	214 (15.2%)		
Other	1,773 (54.0%)	704 (20.0%)		
**Tumor stage**			0.893	0.926
Well-differentiated	1,222 (37.2%)	517 (36.7%)		
Moderately differentiated	896 (27.3%)	402 (28.6%)		
Poorly differentiated	467 (14.2%)	192 (13.6%)		
Undifferentiation	81 (2.5%)	34 (2.40%)		
Other	617 (18.8%)	263 (18.70%)		
**Operation**			0.039	0.843
Yes	3,191 (97.2%)	1,370 (97.3%)		
No	92 (2.80%)	38 (2.7%)		
**Radiotherapy**			0.885	0.347
No	3,245 (98.8%)	1,387 (98.5%)		
Yes	38 (1.2%)	21 (1.5%)		
**Chemotherapy**			1.399	0.237
No	2,238 (68.20%)	935 (66.4%)		
Yes	1,045 (31.8%)	473 (33.6%)		
**Number of lymph node excision**			0.283	0.595
<12	1,749 (53.3%)	762 (54.10%)		
≥12	1,534 (46.7%)	646 (45.90%)		
**Insurance status**			26.167	<0.001
No	137 (4.20%)	110 (7.80%)		
Yes	3,146 (95.80%)	1,298 (92.2%)		
**Marital status**			1.045	0.307
Married	1,920 (58.5%)	846 (60.10%)		
Unmarried	1,363 (41.5%)	562 (39.9%)		
**T stages**			4.066	0.254
T1	978 (29.8%)	421 (29.9%)		
T2	279 (8.5%)	98 (7.0%)		
T3	843 (25.7%)	386 (27.4%)		
T4	1,183 (36.0%)	503 (35.7%)		
**N stages**			0.495	0.781
N0	2,648 (80.7%)	1,124 (79.8%)		
N1	428 (13.0%)	189 (13.4%)		
N2	207 (6.30%)	95 (6.7%)		
**M stages**			3.401	0.065
M0	2,466 (75.10%)	1,093 (77.6%)		
M1	817 (24.9%)	315 (22.4%)		
**Tumor size (CM)**			0.054	0.816
<5	1,991 (60.6%)	1,292 (39.4%)		
≥5	1,292 (39.4%)	549 (39.0%)		
**CEA**			4.838	0.089
Positive	452 (13.8%)	174 (12.4%)		
Negative	449 (13.7%)	169 (12.0%)		
Other	2,382 (72.6%)	1,065 (75.6%)		

### Univariate and multivariate cox regression analysis of the modeling and validation sets

**Figure 1:**
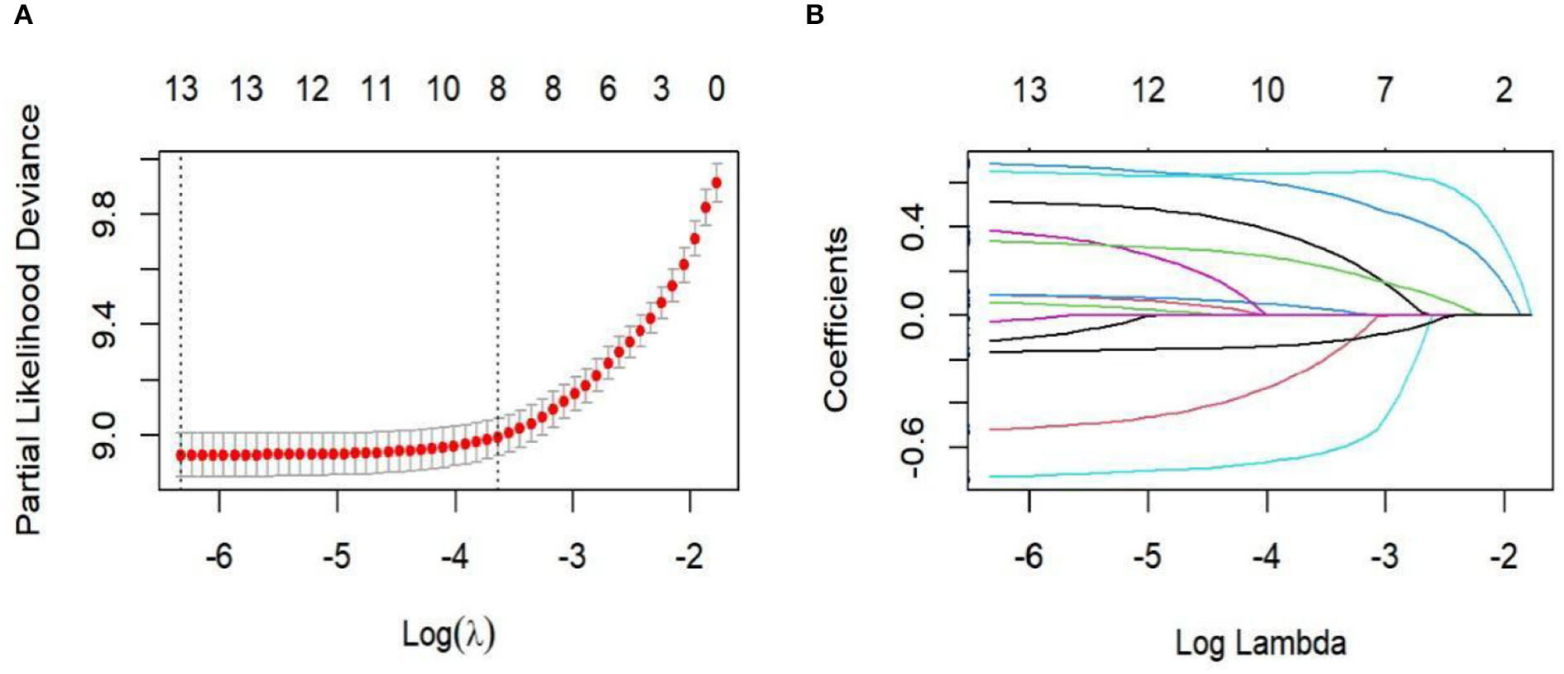
Lasso regression for cross-validation and regression analysis. **(A)** Cross-validation; **(B)** Lasso regression path diagram.

Single-factor Cox regression analysis found there to be statistically significant differences in age, ethnicity, pathological type, pathological stage, surgery, radiotherapy, chemotherapy, lymph node removal, T stage, N stage, M stage, tumor size, and CEA (*p* < 0.05). Lasso regression and cross-validation were performed on 13 statistically significant variables from the aforementioned single-factor Cox regression analysis (Figure 1). The results found race, pathological type, radiotherapy, chemotherapy, and tumor size variables to be excluded. The variables that were finally screened out by Lasso regression—age, pathological stage, surgery, number of lymph node resections, T stage, N stage, M stage, and CEA—were included in the Cox multivariate regression analysis. The results found age, pathological stage, surgery, number of lymph nodes removed, T stage, N stage, M stage, and CEA to be independent risk factors for appendix cancer prognosis (*p* < 0.05; Table 2).

**Table 2:** Univariate and multivariate analysis of appendiceal cancer prognosis.

	**Univariate analysis**		**Multiplicity**	
	**HR (95% CI)**	***P*-value**	**HR (95% CI)**	***P*-value**
**Age**		<0.001		<0.001
<60	Reference		Reference	
≥60	1.891 (1.638–2.182)		1.714 (1.482–1.982)	
**Race**		0.006		
White people	Reference			
Black people	1.293 (1.029–1.624)			
Other	1.366 (1.079–1.729)			
**Sex**		0.056		
Man	Reference			
Woman	0.872 (0.757–1.004)			
**Pathology type**		<0.001		
Carcinoid	Reference			
Cup-shaped cell Carcinoma	3.311 (2.076–5.282)			
Adenocarcinoma	9.857 (6.534–14.872)			
Other	6.598 (4.448–9.788)			
**Tumor stage**		<0.001		<0.001
Well-differentiated	Reference		Reference	
Moderately differentiated	2.601 (2.082–5.282)		1.966 (1.562–2.474)	
Poorly differentiated	6.818 (5.473–8.493)		3.252 (2.552–4.145)	
Undifferentiation	5.427 (3.750–7.855)		2.155 (1.462–3.176)	
Other	2.173 (1.697–2.781)		1.643 (1.272–2.124)	
**Operation**		<0.001		<0.001
Yes	Reference		Reference	
No	0.204 (0.155–0.267)		0.395 (0.292–0.535)	
**Radiotherapy**		<0.001		
No	Reference			
Yes	2.193 (1.407–3.420)			
**Chemotherapy**		<0.001		
No	Reference			
Yes	2.542 (2.207–2.928)			
**Number of lymph node excision**		<0.001		<0.001
<12	Reference		Reference	
≥12	0.830 (0.720–0.956)		0.561 (0.478–0.657)	
**Insurance status**		0.994		
No	Reference			
Yes	0.999 (0.711–1.402)			
**Marital status**		0.146		
Married	Reference			
Unmarried	1.112 (0.964–1.282)			
**T stages**		<0.001		<0.001
T1	Reference		Reference	
T2	1.349 (0.880–2.067)	0.170	1.138 (0.739–1.752)	
T3	2.749 (2.077–3.640)		1.613 (1.198–2.171)	
T4	6.238 (4.824–8.067)		2.304 (1.719–3.088)	
**N stages**		<0.001		<0.001
N0	Reference		Reference	
N1	2.293 (1.915–2.745)		2.009 (1.660–2.432)	
N2	5.911 (4.909–7.118)		2.894 (2.324–3.603)	
**M stages**		<0.001		<0.001
M0	Reference		Reference	
M1	4.081 (3.543–4.701)		1.765 (1.480–2.106)	
**Tumor size (CM)**		<0.001		
<5	Reference			
≥5	1.780 (1.545–2.051)			
**CEA**		<0.001		0.002
Positive	Reference		Reference	
Negative	0.575 (0.465–0.710)		0.837 (0.6721.043)	
Other	0.304 (0.257–0.358)		0.721 (0.6020.865)	

### Establishment and validation of the OS nomogram of appendiceal cancer

**Figure 2:**
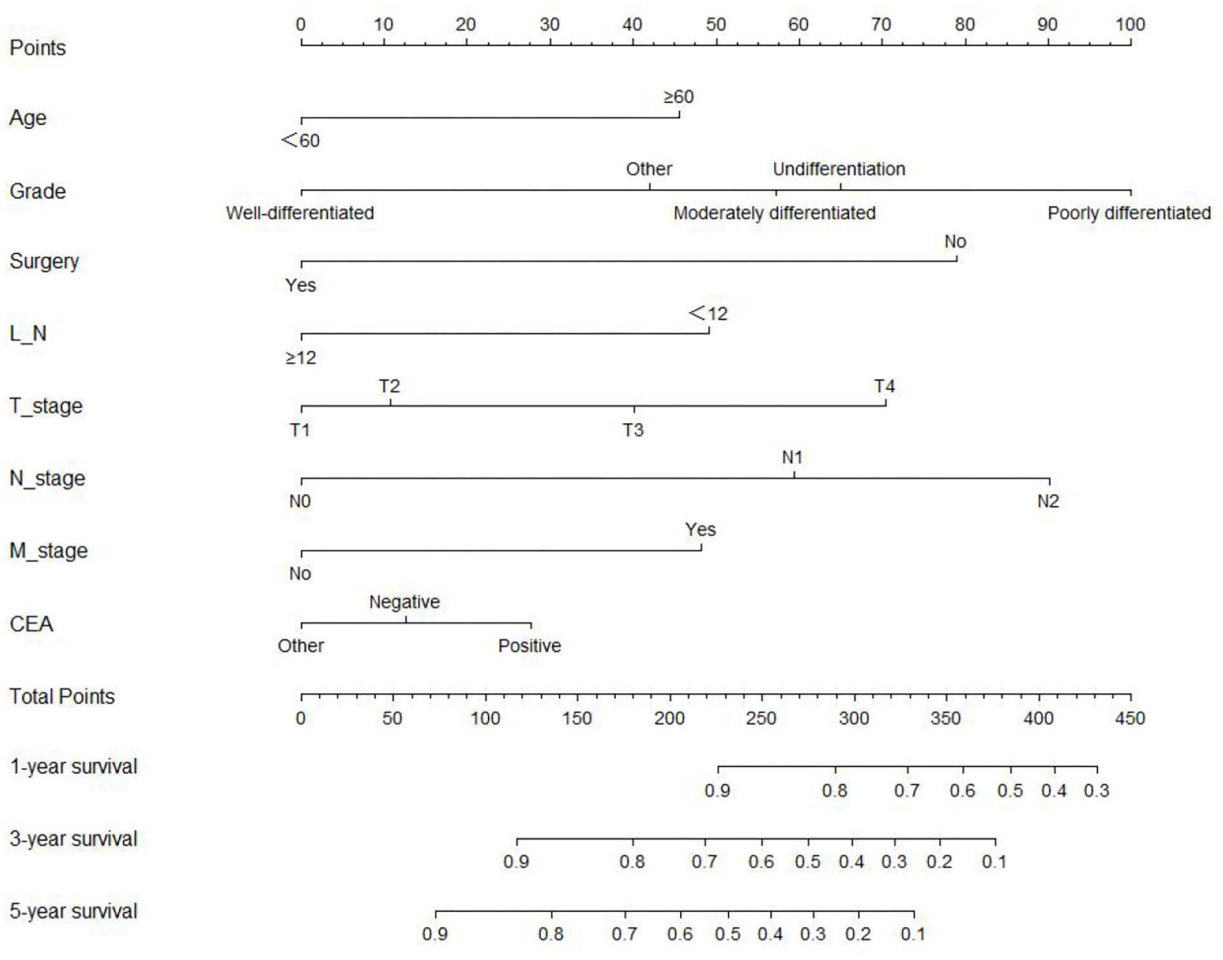
Prediction model of the overall survival (OS) nomogram in appendix cancer patients.

**Figure 3:**
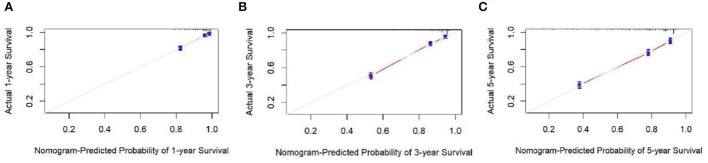
Calibration curves at 1, 3, and 5 years in the modeling set. **(A–C)** Are the calibration curves of modeling sets 1, 3, and 5.

**Figure 4:**
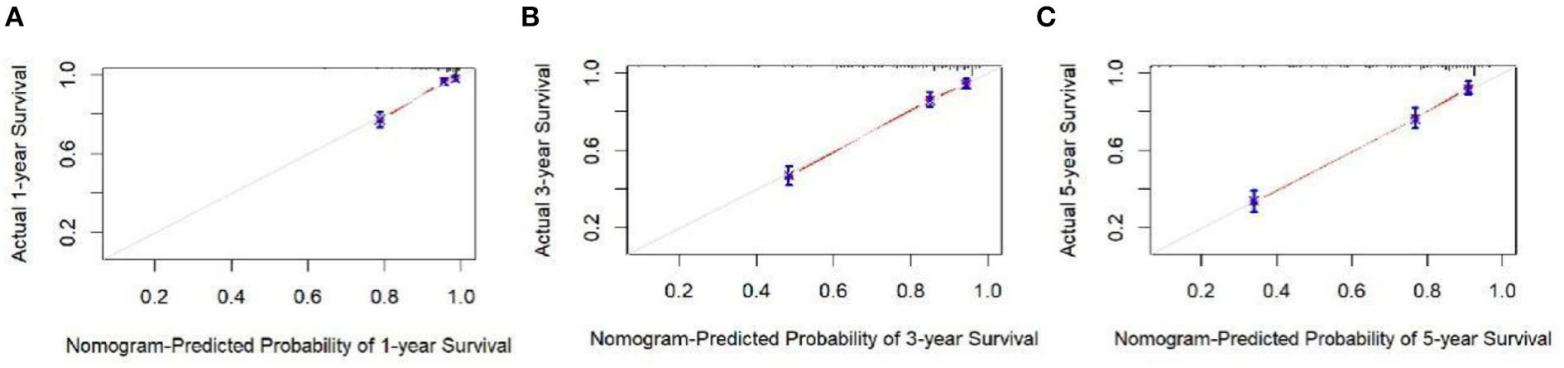
Appendix cancer outcome 1-, 3-, and 5-year calibration curves of validation set patients. **(A–C)** Are calibration curves for validation sets 1, 3, and 5.

**Figure 5:**
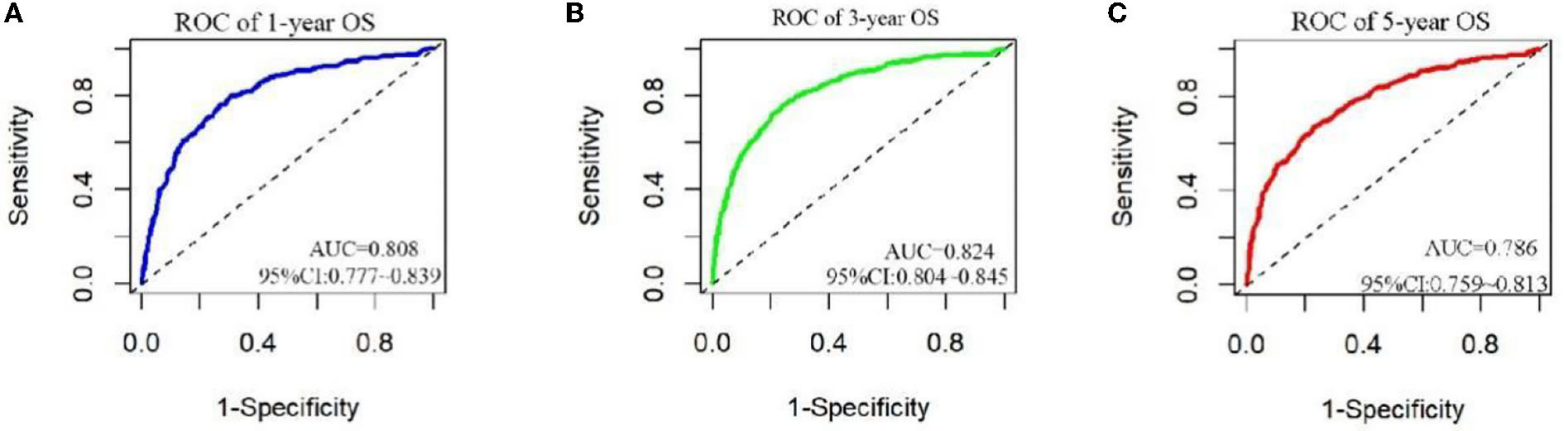
ROC curves for 1, 3, and 5 years in patients. **(A–C)** Are ROC curves for modeling sets 1, 3, and 5.

**Figure 6:**
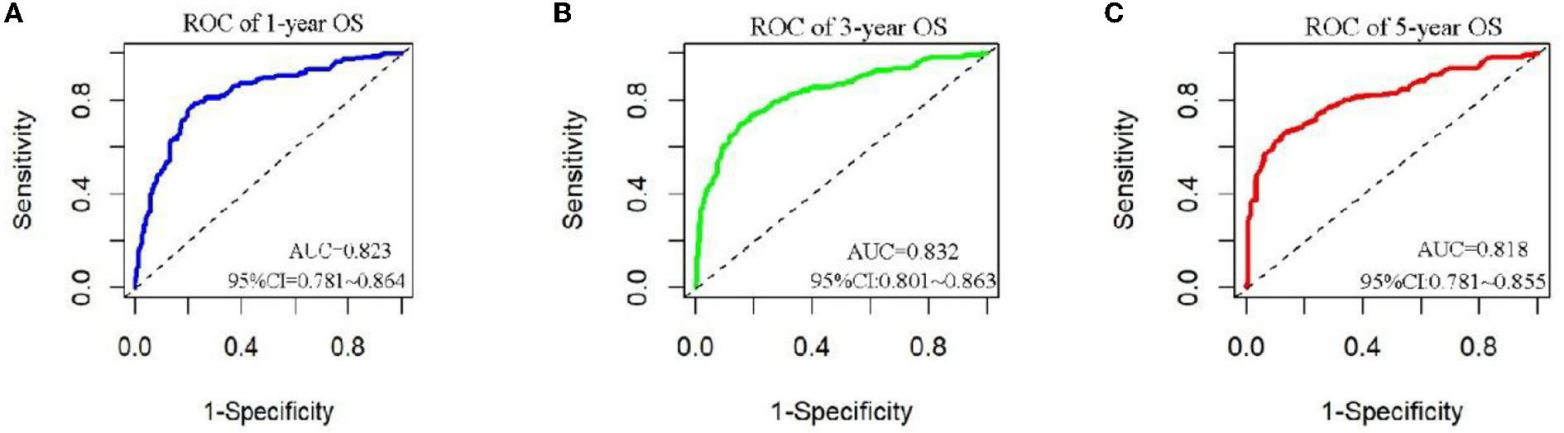
ROC curves for 1, 3, and 5 years of appendix cancer prognosis in validation set patients. **(A–C)** Are ROC curves for validation sets 1, 3, and 5.

**Figure 7:**
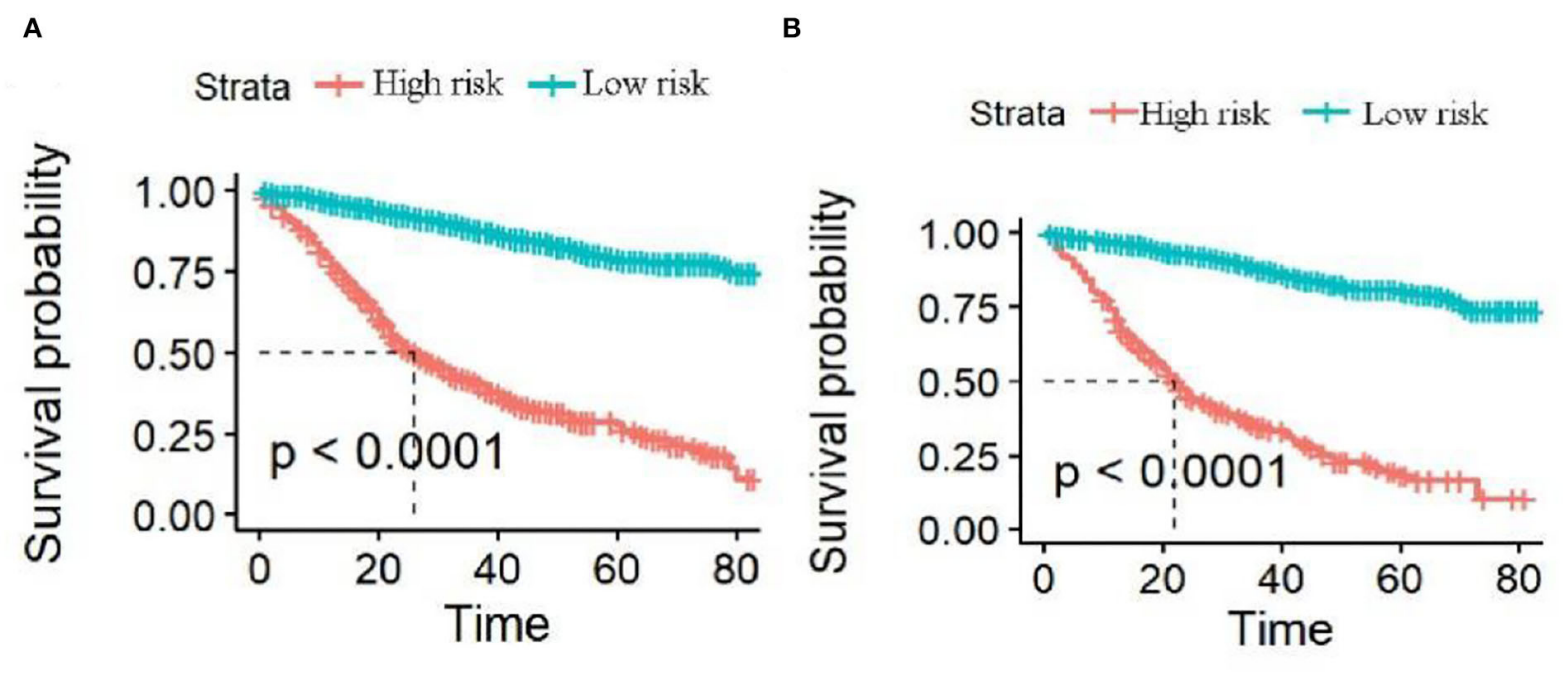
Survival curves of high-risk and low-risk groups predicting 1-, 3-, and 5-year overall survival (OS). **(A,B)** Are the 1-, 3-, and 5-year survival curves of the modeling and validation sets, respectively.

The analysis of significant differences in multifactorial Cox regression was incorporated into the R software, and a nomogram model of OS that affects appendiceal cancer was established (Figure 2) for predicting 1-, 3-, and 5-year OS in appendiceal cancer patients. The internal validation of the calibration curve found there to be good agreement between 1-, 3-, and 5-year OS predicted by the model and actual OS (Figures 3, 4). In the 1-, 3-, and 5-year modeling sets, the areas under the ROC curve were 0.808 (95% CI: 0.777–0.839), 0.824 (95% CI: 0.804–0.845), and 0.786 (95% CI: 0.759–0.813; Figure 5). The areas under the ROC curve for the 1-, 3-, and 5-year validation sets were 0.823 (95% CI: 0.781–0.864), 0.832 (95% CI: 0.801–0.863), and 0.817 (95% CI: 0.781–0.855; Figure 6). Finally, probabilistic values were calculated based on multifactorial Cox risk regression, and the optimal cut-off values that correspond to the maximum Jordan index of the ROC curve were divided into high-risk and low-risk groups. The Kaplan–Meier survival curve showed the 1-year survival rate in the modeling cohort to be 67.3%, the 3-year specific survival rate was 19.8%, and the 5-year specific survival rate was 3.1%. The validation set had a 1-year survival rate of 64.0%, a 3-year specific survival rate of 17.9%, and a 5-year specific survival rate of 3.4%. The results from the modeling and validation sets were found to be consistent, with the high-risk groups having poor prognoses and the low-risk groups having better prognoses (*p* < 0.001; Figure 7).

## Discussion

Appendiceal cancer is a rare tumor that is found in the digestive tract, and it is often reported on a case-by-case basis. The diagnosis of appendiceal cancer is currently based mostly on postoperative pathology. There are various appendiceal cancer clinical symptoms. During the early stage, no obvious clinical symptoms or pain may be evident at McBurby's point. During the late stage, symptoms including intestinal obstruction and ascites may occur. Appendiceal cancer is often incorrectly clinically diagnosed as acute appendicitis or ovarian adnexal-derived tumors (3). As a preoperative colonoscopy only shows the mucosa of the colorectum, it is impossible to take a biopsy of the appendix mucosa, which is decidedly unhelpful when diagnosing appendix cancer. In the blood biochemical examination, the CEA tumor marker may exhibit an increase, which suggests that it is derived from the digestive tract. Appendiceal cancer advances slowly and good results are generally achieved with surgery. Among domestic and foreign studies, those relevant to appendix cancer are mainly case reports, small sample sizes, and single-center studies. They have low credibility, the selection of variables is mainly subjective, and objective evaluation is lacking. Using data from the SEER database, Lasso regression was used for screening the best variables, establishing a nomogram model, reducing model bias, performing multivariate Cox regression analysis of appendix cancer clinical-pathological data and independent risk factors that affect appendix cancer OS, and establishing an appendix cancer prognosis model for providing a certain clinical basis for appendix cancer survival prognosis.

Decreased pain sensitivity among elderly patients may lead to the promotion of the progression of local cancer tissue and metastasis of distant organs, which results in missed optimal treatment time and reduced survival time (4). The degree of differentiation of appendix cancer cells directly reflects the degree of tumor malignancy through the heterogeneity of tumor cells and mitotic images. According to relevant reports (5), the pathological stage of the tumor is linked to the development of anemia in the body, which affects the patient prognosis. Therefore, this may a reason why the pathotype of highly differentiated appendices has a better prognosis than medium and low-differentiated appendices. This study found that compared to non-surgical patients (HR = 0.395 95% CI: 0.292–0.535), the surgical patient prognosis was significantly higher than non-surgical patient prognosis. Therefore, it is recommended that appendix cancer patients actively undergo radical surgical treatment. However, a certain amount of controversy remains regarding surgical treatment methods. It is considered that (6) patients with appendiceal cancer with a tumor >2 cm, late T stage and N stage, and positive suspicious margins should undergo an appendectomy in combination with right hemicolectomy as a means of reducing the local recurrence of the tumor. Another study found that (7) the removal of the primary lesion of appendix cancer alone can achieve good results, and when combined with right hemicolectomy, the OS time of appendix cancer is not improved. This conclusion still requires further verification through a series of multicenter studies or higher-level META analysis studies. It was determined that extensive resection should be performed for radical treatment purposes, regardless of tumor stage, in order to reduce lymphatic metastasis of appendix cancer and reduce the probability of recurrence following surgery. Lymph node metastasis has been identified as an independent risk factor for the prognosis of the gastric, colon, and other gastrointestinal cancers (8–10). According to a research report (11), ~38.4% of patients who are diagnosed with appendiceal cancer have metastatic lesions, so a focus on lymph node metastasis is of the utmost importance for patient prognosis. In this study, it has been shown that the radical surgical removal of ≥12 lymph nodes is a protective factor that affects appendectomy prognosis, which is consistent with the findings of Fleischmann (11). Therefore, the radical surgical removal of ≥12 lymph nodes is recommended for appendectomy patients as a means of improving survival as much as possible. The study suggests (12) that hyperthermic intraperitoneal chemotherapy (HIPEC) is recommended for patients with mucinous adenocarcinoma of the appendix. However, no consensus on neoadjuvant chemotherapy + surgery, surgery + postoperative adjuvant chemotherapy, radical surgery only, or other targeted and immunotherapy currently exists for appendiceal cancer treatment, and further prospective studies are required. TNM staging has long been regarded as a pivotal indicator for the assessment of treatment means and oncological outcomes (13, 14). The results of this study reveal that the later the stage, the higher the risk ratio (HR), and the later the stage indicates that the deeper the tumor invasion of the intestinal wall, the greater the chance of vascular and nerve invasion and the increased probability of metastasis in the distant organs of the tumor cells. CEA is a gastrointestinal cancer marker that is involved in disease diagnosis and the evaluation of disease prognosis (15–17). Several previous studies have found CEA positivity to be a poor prognostic factor in the gastrointestinal tract (18), which is consistent with the results of this study.

The nomogram model quantifies and visualizes the Cox risk regression results of disease prognosis and is currently widely used in liver, breast, and kidney cancer (19–21). Based on the multifactorial Cox risk regression analysis results, a nomogram model was constructed to predict appendiceal cancer 1-, 3-, and 5-year survival rates, and appendiceal cancer patients were classified into high- and low-risk groups with optimal cut-off values. The results found the modeling and validation sets to be consistent, with a difference in survival time for high-risk groups and a better prognosis for low-risk groups. Age, pathological stage, surgery, number of lymph nodes removed, T stage, N stage, M stage, and CEA were also identified as independent risk factors in appendix cancer prognosis. Therefore, attention should be paid to these indicators in clinical practice, high-risk and low-risk patients should be distinguished between, and they should be provided with personalized treatment plans.

In this study, the SEER database was modeled, and an internal verification model was established. ROC curves and calibration curves were used for evaluating the model, and the results were found to be relatively good. However, due to the rarity of appendectomies, an external validation set for better evaluating the reliability of the model could not be established. Furthermore, the database was unable to obtain vascular nerve infiltration and could not be analyzed in more depth, so there are limitations to the study.

In conclusion, age, pathological stage, surgery, lymphadenectomy number, T stage, N stage, M stage, and CEA are independent risk factors that affect appendix cancer prognosis. The nomogram model that is based on these indicators has good predictive value for1-, 3-, and 5-year survival rates. At the same time, the aforementioned factors should be considered for the detection of appendix cancer with early intervention among high-risk groups.

## Data availability statement

The raw data supporting the conclusions of this article will be made available by the authors, without undue reservation.

## Ethics statement

The authors were authorized to extract data from the SEER study by the National Cancer Institute. Access to data *via* the SEER database requires no informed patient consent (SEER ID: 13846—Nov2020). This study is a retrospective analysis that strictly complies with the Helsinki Declaration of 1964 and any subsequent amendments or similar ethical standards.

## Author contributions

TL designed, analyzed, and wrote the paper. JM and YW participated in the statistical analysis and revised the paper. WQ, CW, and ZM participated in arranging the data. CW were responsible for finalizing the manuscript. All authors agree with the above contributions. All authors contributed to the article and approved the submitted version.

## Funding

The study was supported by the National Natural Science Foundation of China (82060485), the Natural Science Foundation project of Qinghai Provincial Science and Technology Department (2017-ZJ-921Q), and Supported by Medical Science Research Foundation (YWJKJJHKYJJ-YWKYQ2003).

## Conflict of interest

The authors declare that the research was conducted in the absence of any commercial or financial relationships that could be construed as a potential conflict of interest.

## Publisher's note

All claims expressed in this article are solely those of the authors and do not necessarily represent those of their affiliated organizations, or those of the publisher, the editors and the reviewers. Any product that may be evaluated in this article, or claim that may be made by its manufacturer, is not guaranteed or endorsed by the publisher.
